# Septin cooperation with tubulin polyglutamylation contributes to cancer cell adaptation to taxanes

**DOI:** 10.18632/oncotarget.5373

**Published:** 2015-10-07

**Authors:** Laurence Froidevaux-Klipfel, Benjamin Targa, Isabelle Cantaloube, Hayat Ahmed-Zaïd, Christian Poüs, Anita Baillet

**Affiliations:** ^1^ INSERM, UMR-S-1193, Université Paris-Saclay, Châtenay-Malabry, France; ^2^ Laboratoire de Biochimie-Hormonologie, Hôpital Antoine Béclère, AP-HP, Clamart, France

**Keywords:** septin, microtubule polyglutamylation, Taxol^®^ resistance, tyrosinated tubulin

## Abstract

The mechanisms of cancer cell adaptation to the anti-microtubule agents of the taxane family are multifaceted and still poorly understood. Here, in a model of breast cancer cells which display amplified microtubule dynamics to resist Taxol^®^, we provide evidence that septin filaments containing high levels of SEPT9_i1 bind to microtubules in a way that requires tubulin long chain polyglutamylation. Reciprocally, septin filaments provide a scaffold for elongating and trimming polyglutamylation enzymes to finely tune the glutamate side-chain length on microtubules to an optimal level. We also demonstrate that tubulin retyrosination and/or a high level of tyrosinated tubulin is crucial to allow the interplay between septins and polyglutamylation on microtubules and that together, these modifications result in an enhanced CLIP-170 and MCAK recruitment to microtubules. Finally, the inhibition of tubulin retyrosination, septins, tubulin long chain polyglutamylation or of both CLIP-170 and MCAK allows the restoration of cell sensitivity to taxanes, providing evidence for a new integrated mechanism of resistance.

## INTRODUCTION

Despite Taxol^®^ success in chemotherapy of advanced breast cancer, acquired drug resistance limits the long-term effectiveness of this microtubule (MT)-targeting agent [1] and contributes in making breast cancer the second leading cause of cancer death in women. Paclitaxel (Taxol^®^) binding to β-tubulin and subsequent alteration of MT dynamics and mitosis completion leads to apoptosis, but several mechanisms of resistance have been described (for reviews, see McGrogan et al. [1] and Murray et al. [2]), which comprise the multidrug-resistance phenotype mediated by the *P*-glycoprotein efflux pump and a variety of adaptive mechanisms that affect MT dynamics like β-tubulin mutations, altered expression of tubulin isotypes or of MT-associated proteins (MAPs). We previously found that several septins (SEPT2, 8, 9 and 11) are overexpressed in Taxol^®^-resistant MDA-MB 231 breast cancer cells and preferentially associated with MTs [3].

Septins are a family of GTP-binding proteins forming apolar hetero-oligomeric filaments that assemble into higher-order structures associated with actin microfilaments or with MTs [4–6]. Septins have emerged as multifunctional scaffolding proteins and organizers of membrane diffusion barriers [7–9]. They are implicated in a growing array of functions, including cytokinesis [10, 11], migration [12, 13], vesicular transport [14], ciliogenesis [15], bacterial internalization [16] but also in neurological diseases [17, 18] and in a multitude of cancers (for review, see Russell and Hall [19]). In mammals, among the 13 septin genes that have been identified [20, 21], SEPT9 has already been proposed to participate in the resistance to MT-disrupting agents through a mechanism involving HIF-1α [22]. It also is the only septin in which specific repeated motifs might allow MT binding and bundling [23]. Understanding how septins could be involved in cell adaptation to anticancer drug-mediated alterations of MT dynamics is challenging because they may interfere with tubulin modifications frequently encountered in long-lived MTs like polyglutamylation [14]. Tubulin polyglutamylation consists in the branching and elongation (up to 6 residues) of lateral peptide chains of glutamate residues near the C-terminus of α- and/or β-tubulin. Polyglutamylation plays an important role in modulating MT stability by controlling the association of MAPs like tau and MAP2 [24] or the binding of the MT-severing enzymes katanin and spastin [25, 26] and was implicated in neurodegenerative disorders [27]. The detyrosination/tyrosination cycle of α-tubulin was associated with tumor aggressiveness [28] and Taxol^®^ resistance of breast cancer cells [29]. Indeed, accumulation of detyrosinated α-tubulin (hereafter named Detyr-tubulin) enhances MT stability by preventing the binding to MTs of depolymerases like MCAK [30], and of the rescue factor CLIP-170 [31]. Conversely, it favors spastin-mediated severing of MTs [32] and is involved in the binding and motor activity of kinesin-1 [33, 34]. Here, we provide evidence that septin binding to tyrosinated and polyglutamylated MTs plays a scaffolding role in controlling the length of polyglutamate chains. Altogether, septins and these tubulin modifications result in the modulation of the recruitment of catastrophe and rescue factors that would in turn allow the restoration of high levels of MT dynamic instability, contributing to the Taxol^®^-resistant phenotype.

## RESULTS

### Taxol^®^-adapted cells display a high level of microtubule dynamics

**Figure 1:**
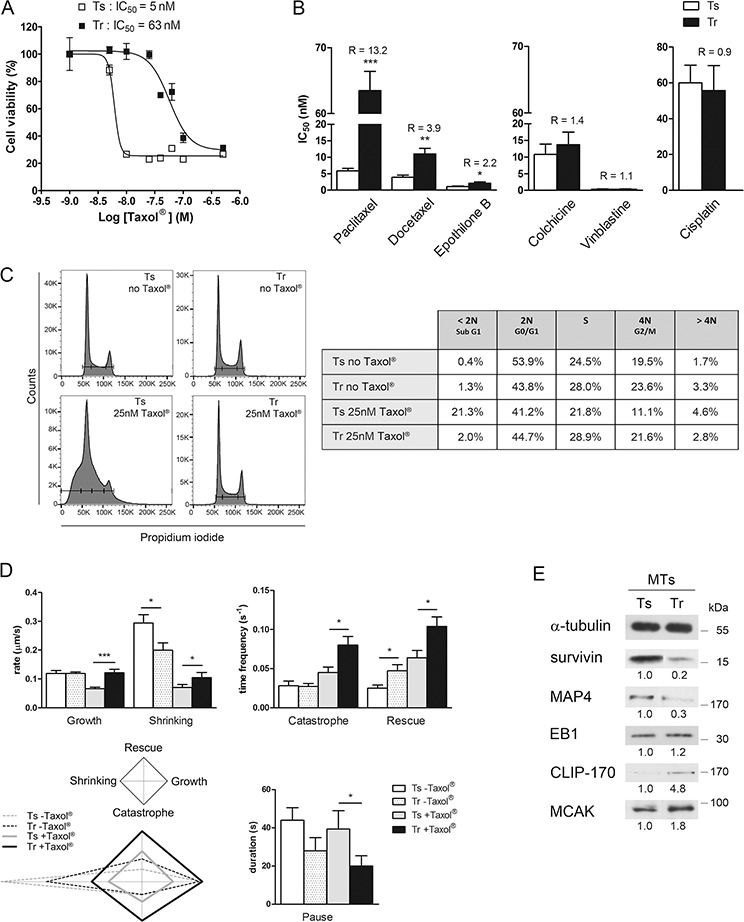
Paclitaxel-resistant MDA-MB 231 cells exhibit a high level of microtubule dynamics **A.** Tr cells resist Taxol^®^. Cell viability of Ts and Tr cells was determined by MTT assay after 48 h exposure to increasing concentrations of Taxol^®^. The graph shows the data of one representative experiment out of at least twenty independent IC_50_ determinations. **B.** Tr cells resist taxanes and epothilone B, but not other MT-targeting drugs or cisplatin. R values are the IC_50_ ratios of Tr to Ts mean values. **C.** Tr cells are fully adapted to Taxol^®^. Cell cycle analysis was performed after treatment with or without 25 nM Taxol^®^ for 24 h. **D.** MT dynamics are amplified in Tr cells. Mean ± s.e.m values of the dynamic instability parameters were measured from Ts and Tr cells (at least 8 MTs from 3 cells in each condition) after treatment with 25 nM Taxol^®^ for 24 h (histograms) and plotted as diamond graphs for easier global comparison. **E.** The MT fractions of Ts and Tr cells exhibit different amounts of MAPs; α-tubulin was used as the loading control.

Taxol^®^-resistant (Tr) MDA-MB 231 breast cancer cells were previously selected by exposure of the Taxol^®^-sensitive (Ts) cells to incremental Taxol^®^ concentrations [3]. In Tr cells, the IC_50_ of Taxol^®^ is ~13-fold higher than in Ts cells as determined by an MTT assay (Fig. 1A). Cross-resistance of Tr cells was also determined for a variety of MT-targeting drugs (Fig. 1B) and revealed they also resist docetaxel (Taxotere^®^) and to a lesser extent epothilone B (another MT stabilizing drug that binds tubulin near the taxane binding site [35]), indicating a more general resistance to MT-stabilizing drugs. In contrast, Tr cells were sensitive to colchicine and vinblastine, (two MT destabilizers that bind distinct sites on tubulin). The DNA crosslinker cisplatin was used as a negative control. Therefore, Tr cells resist MT stabilizers and more predominantly taxanes (Taxol^®^ more than Taxotere^®^).

Ts cell sensitivity to Taxol^®^ (25 nM, 24 h) was evidenced by measuring their DNA content. As already reported at low doses [36], Taxol^®^ caused Ts cell accumulation in a subG1 peak that could not be entirely resolved from the G0/G1 one, and which suggested the accumulation of apoptotic cells without cell cycle arrest. In contrast, Tr cells cultured with or without Taxol^®^ (25 nM, 24 h) exhibited very similar populations in G0/G1, in G2/M and in S phase (Fig. 1C). These results show that Tr cells, which are continuously cultured in the presence of 25 nM Taxol^®^, are fully adapted to the drug.

To determine whether Tr cell adaptation to Taxol^®^ actually involved a modulation of MT dynamics, we next measured the parameters of MT dynamic instability of Ts and Tr cells after a 24 h exposure to 25 nM Taxol^®^ compared to untreated Ts and Tr (Fig. 1D). Dynamic instability exhibited significant increase in all the parameters measured in the Tr+Taxol^®^ *vs* Ts+Taxol^®^ conditions, except for the duration of pauses that was shorter. From this set of data, we constructed the diamond graphs shown in Fig. 1D (left bottom panel, as described by Lacroix et al. [37]), in which the vertical elongation of the shape reflects high transition frequencies and the horizontal elongation, the speed of MT length variation. The shape of the diamonds is affected in a very similar way in Ts and Tr cells by the presence of Taxol^®^ with higher transition frequencies and slower length variation, but in Tr cells, the four parameters are amplified. This, together with the shorter pause duration, indicates that Tr cells actually adapted their MT dynamics to compensate for the effects of Taxol^®^. Further analysis of the MT fractions of Ts and Tr cells (Fig. 1E) allowed us to identify several modifications in the level of MT regulators. Indeed, survivin, which is an apoptotic inhibitor but also a stabilizer of MTs [38], was less abundant in the MT fraction of Tr cells. Similarly, a reduced level of MAP4 (which stabilizes MTs) was observed in Tr compared to Ts cells. Regarding plus end Tracking Proteins (+TIPs), EB1 level was roughly unchanged in Tr *vs* Ts but the rescue factor CLIP-170 and the depolymerizing kinesin MCAK were more abundant in the MT fraction of Tr cells. Altogether, these data indicate that Tr cells, which are continuously cultured in the presence of 25 nM Taxol^®^ display enhanced MT dynamics that could be related to alterations in the recruitment of MT regulators, and which would in turn compensate for the stabilizing effect of taxanes.

### Increased septin recruitment to microtubules is required for Taxol^®^ resistance

**Figure 2:**
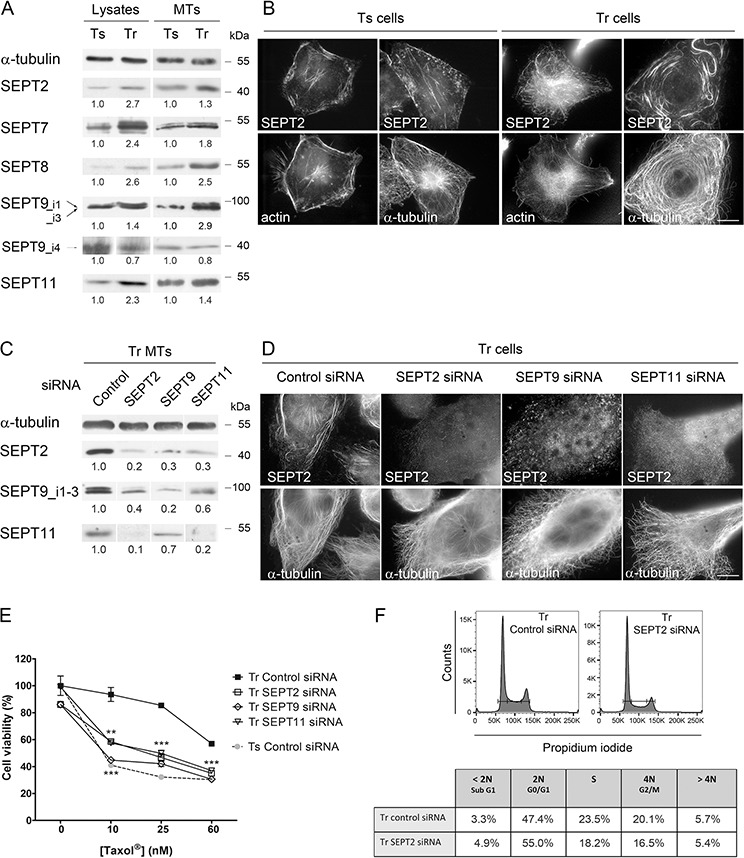
Increased recruitment of septins to microtubules confers Taxol^®^ resistance **A.** Patterns of septin whole cell expression (Lysates) and recruitment to MTs in Tr *vs* Ts cells. A constant protein (20 μg for Lysates) or α-tubulin amount (for MT fractions) was loaded on gels for each sample. **B.** SEPT2 localization switches from F-actin in Ts to MTs in Tr cells. Scale bar = 10 μm. **C.** Knockdown of individual septins affects the presence of other septins in the MT fraction of Tr cells. **D.** Individual septin inhibition alters the formation of septin filaments and their localization to MTs in Tr cells. **E.** Individual septin inhibition by RNAi restores chemosensitivity of Tr cells to Taxol^®^ as determined by MTT assay. **F.** SEPT2 depletion (72 h RNAi) did not cause the accumulation of Tr cells in G2/M.

**Figure 3:**
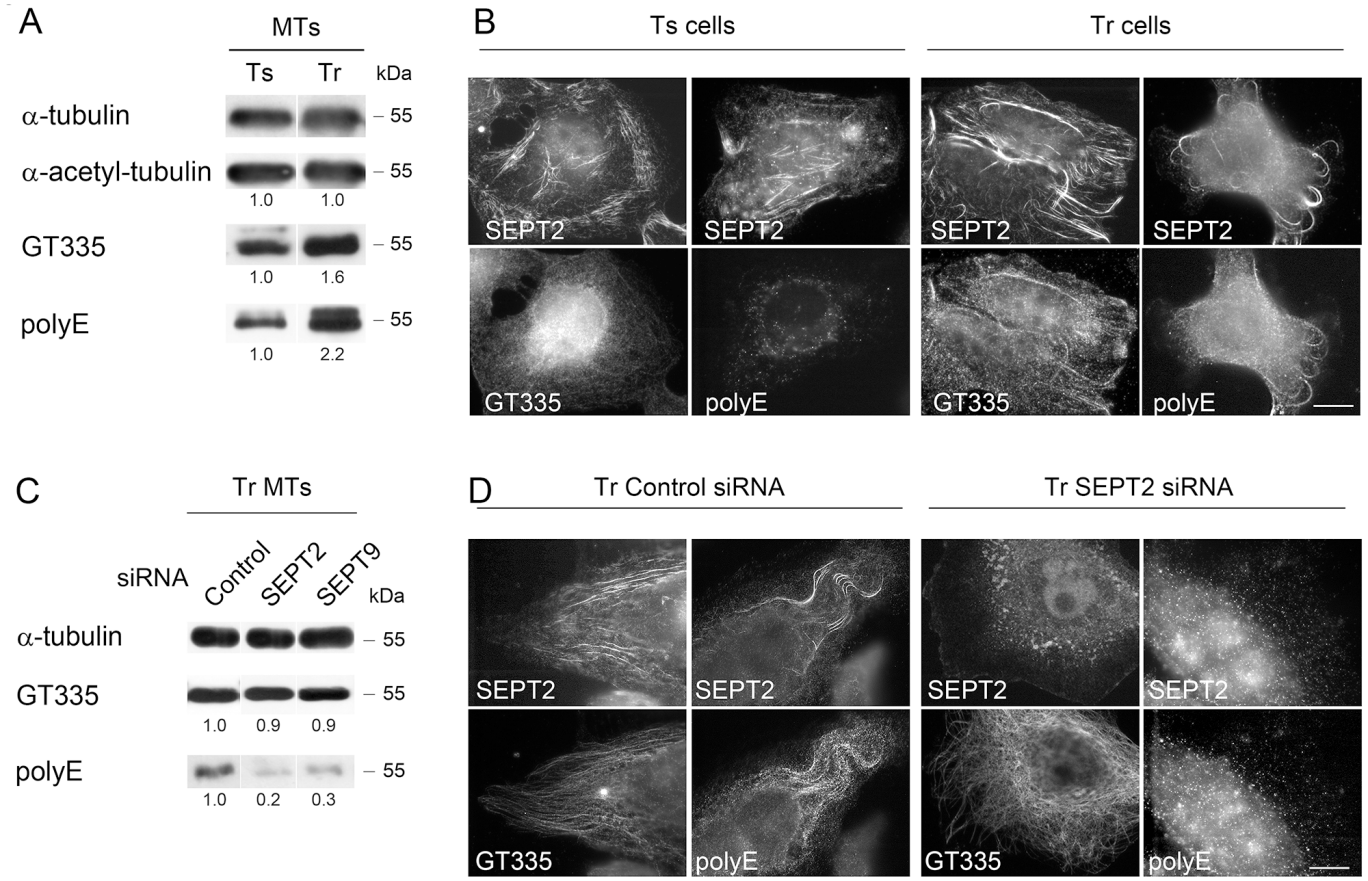
Septins are required to allow long chain polyglutamylation of microtubules in Tr cells **A.** MTs of Tr cells are highly polyglutamylated. The GT335 antibody was used to detect the polyglutamate lateral chains of any length while the polyE antibody is selective of long chains. **B.** Ts cells exhibit low levels of long chain-polyglutamylated tubulin that do not colocalize with SEPT2 labeling. Conversely, septins localize to MTs bearing long polyglutamate chains in Tr cells. Scale bar = 10 μm. **C** and **D.** SEPT2 or SEPT9 knockdown by RNAi prevents long polyglutamate chain formation on the MTs of Tr cells.

As Western-blotting of SEPT2, 7, 8, 9 and 11 confirmed their overexpression and higher recruitment in the MT fraction of Tr cells compared to Ts (Fig. 2A and [3]), we next tried to understand how septins are involved in the changes that affect MTs in Tr cells. A more detailed analysis of SEPT9 isoforms revealed that among the long isoforms of the protein (SEPT9_i1 and SEPT9_i3), SEPT9_i3 was predominantly detected in Ts cells. Conversely, SEPT9_i1, which was already proposed to participate in Taxol^®^ resistance [22], was highly expressed in Tr cells and enriched in their MT fraction (Fig. 2A). By contrast, SEPT9_i4, which is a shorter isoform overexpressed in certain breast and ovary cancer cells [39], was downregulated and less abundant in the MT fraction of Tr cells. In accordance with the higher recruitment of SEPT2, 7, 8, 9_i1, 9_i3 and 11 in the MT fractions of Tr cells, SEPT2-labelled filaments predominantly relocalized from cortical actin and stress fibers in Ts cells to a population of MTs in Tr cells (Fig. 2B). Septins are known to assemble into heterotrimers or heterotetramers that include one protein from each of the four septin groups in a precise order: SEPT2, SEPT6 (which can be replaced by SEPT8 or 11), SEPT7 and SEPT9 [40, 41]. These hetero-oligomers that are arranged as perfect palindromes (thus forming hexamers or octamers) associate to form nonpolar filaments [42]. Due to this arrangement, the depletion of SEPT2 or SEPT7 may cause the degradation of other septins [4, 42]. This is why, when cells were depleted of SEPT2, 9 or 11 by RNAi, the expression and MT recruitment of septins from all the groups was impaired (Fig. 2C) and septin filaments could no longer be detected in depleted cells (Fig. 2D). Therefore, in the rest of the study, RNAi depletion of either septin was interchangeably applied to totally disorganize the septin filament network and perturb the overall septin function.

To determine the role played by septins in chemoresistance, Tr cells were challenged with increasing Taxol^®^ concentrations (10, 25 and 60 nM i.e. twice the IC_50_ of Taxol^®^ in Ts cells, the concentration used in Tr cell culture and the IC_50_ of Taxol^®^ in Tr cells, respectively) (Fig. 2E). After depletion of SEPT2, 9 or 11 by RNAi, Tr sensitivity to Taxol^®^ was restored to almost the same level as that measured in Ts cells. While septins were primarily described for their crucial role in cytokinesis [43], their depletion did not cause a blockade in the G2/M phase in Tr cells (Fig. 2F). The reversion of Taxol^®^ resistance we observed might thus have resulted from septin loss from MTs.

### The microtubules of Taxol^®^-resistant cells display septin-dependent long chain polyglutamylation

Since septins were formerly shown to associate with polyglutamylated MTs specialized in vesicular trafficking [14], we tested whether this post-translational modification of tubulin was important for septin recruitment to MTs in Tr cells. MTs of Tr cells exhibited similar acetylated but higher levels of polyglutamylated tubulin than Ts MTs as evidenced by the higher signals obtained with the GT335 and the polyE antibodies, which reveal polyglutamate chain branching on tubulin and long (≥ 3 units) polyglutamate chains, respectively (Fig. 3A and 3B). Immunofluorescence experiments further revealed that highly polyglutamylated MTs (in particular long polyglutamylated chains) colocalize with SEPT2 in Tr cells (Fig. 3B). Two-dimensional electrophoresis showed accordingly that both α- and β-tubulin of Tr cells display long polyglutamylated chains (Fig. S1A). As a control, we checked that the amount of long glutamate side-chains did not increase upon acute Taxol^®^ treatment (Fig. S1B). Similarly, septins were not massively relocalized to MTs in Ts cells treated with 25 nM Taxol^®^ for 24 h, as compared to Tr cells (Fig. S1B and S1C). Thus, both higher polyglutamylation level and septin recruitment to the MTs of Tr cells reflect a long-term adaptive mechanism to Taxol^®^.

As septins were proposed to be required for the maintenance of polyglutamylated MTs [14], we further examined the impact of septin depletion on the level of tubulin polyglutamylation in the MTs of Tr cells (Fig. 3C and 3D). Strikingly, SEPT2 or SEPT9 depletion did not affect the GT335 signal but they strongly decreased the polyE one (Fig. 3C and 3D). In parallel, the depletion of SEPT2 resulted in the specific loss of MT-shaped polyE signal organization, while GT335 labeling still revealed MTs (Fig. 3D). Therefore, the overall tubulin polyglutamylation only partly depends on septin recruitment to the MT lattice. Nevertheless, septins are important for the control of polyglutamate chain length.

### Long polyglutamate chains favor septin recruitment to microtubules and are also required for Taxol^®^ resistance

**Figure 4:**
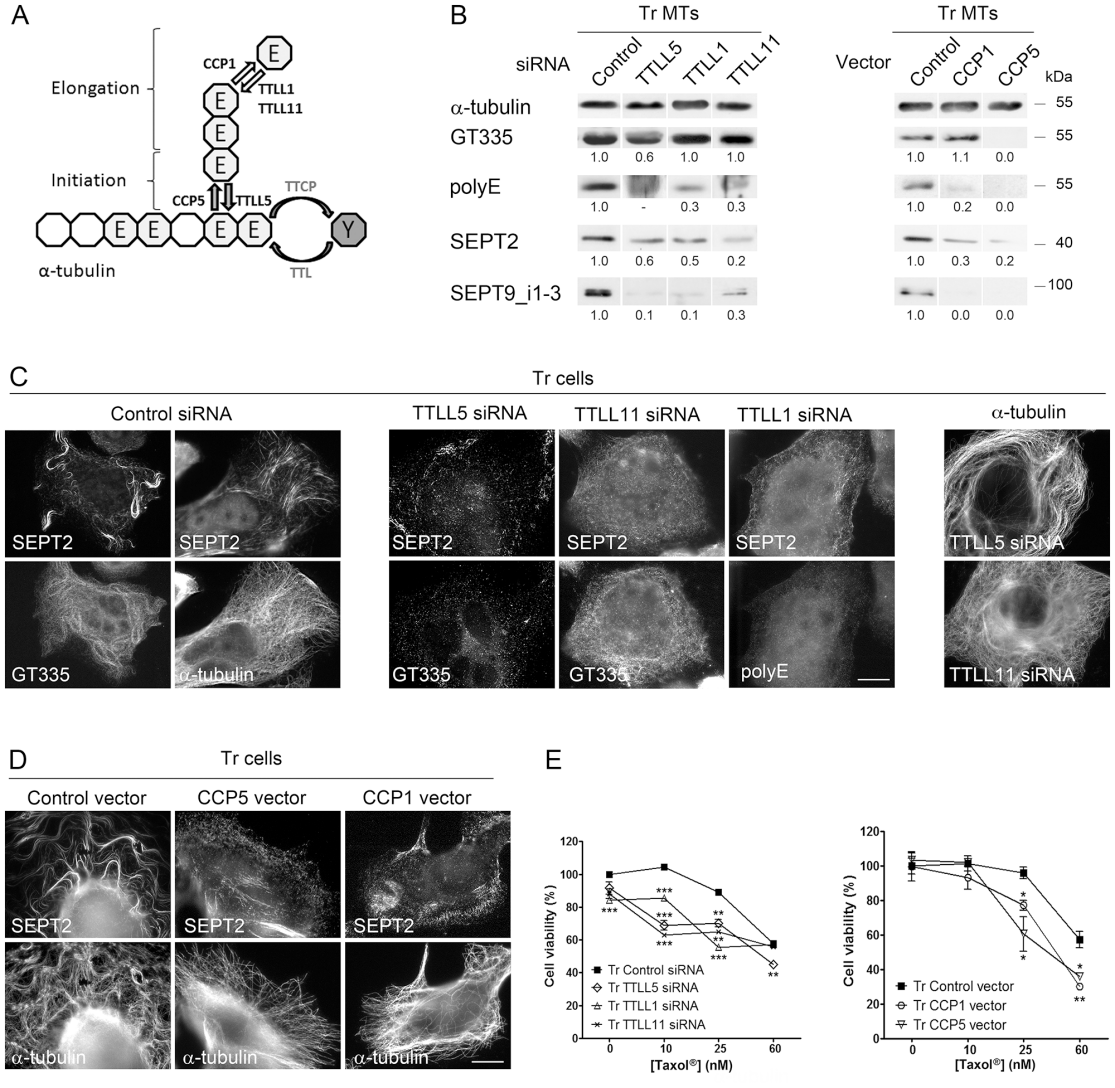
Long polyglutamate chains on tubulin favor septin recruitment to microtubules and participate in Taxol^®^ resistance **A.** Schematic representation (modified from Wloga and Gaertig [83]) of α-tubulin C-terminus with the enzymes responsible for tubulin retyrosination (TTL), polyglutamate chain initiation (TTLL5), elongation (TTLL1 and TTLL11), trimming (CCP1) and debranching (CCP5) used in this project. **B.** Septin recruitment to MTs of Tr cells is altered by the inhibition of polyglutamylation enzymes (Left) and by the overexpression of deglutamylases (Right). Tubulin polyglutamylation and septin recruitment were analyzed on the MT fractions of Tr cells that were transfected as indicated. **C.** Septin filaments are absent from MTs of Tr cells after the inhibition of tubulin polyglutamylation by RNAi of various TTLLs (as detected by the GT335 and/or the polyE antibodies) (Left and central panels). Inhibiting global or long chain polyglutamylation does not affect the MT network (Right panel). Scale bar = 10 μm. **D.** CCP overexpression causes septin filament disorganization without affecting the MT network. **E.** Loss of tubulin polyglutamylation by TTLL RNAi or CCP overexpression in Tr cells partly restores sensitivity to Taxol^®^. Data are the mean ± s.e.m. of 4 measurements after cell exposure to increasing Taxol^®^ concentrations for 48 h.

As tubulin post-translational modifications are expected to modulate the binding of MAPs to the MT surface [44], we further asked whether polyglutamylation could conversely affect septin recruitment to the MTs of Tr cells. Tubulin polyglutamylation consists in two sequential steps of initiation and elongation of glutamate chains near the C-terminus of α- or β-tubulin, each step being catalyzed by specific enzymes of the tubulin tyrosine ligase-like (TTLL) family. TTLLs are highly homologous to tubulin tyrosine ligase (TTL), which re-tyrosinates the C-terminal Glu residue of Detyr tubulin ([45] and Fig. 4A). Symmetrically, polyglutamate chain reduction involves trimming carboxypeptidases like CCP1, which shorten glutamate chains, and debranching enzymes like CCP5. We performed RNAi depletion of the initiating polyglutamylase TTLL5 or of two elongating enzymes TTLL1 (for which overexpression could be evidenced in Tr cells by qPCR as shown in Fig. S2) or TTLL11. As expected, knocking down TTLL5 resulted in an overall decrease in MT polyglutamylation as revealed by reduced GT335 and polyE signals, but also in a drop in SEPT2 and SEPT9_i1–3 recruitment to the MTs of Tr cells (Fig. 4B). While MTs were still present (Fig. 4C), a marked disorganization of septin filaments occurred that followed the loss of global MT polyglutamylation as revealed by the drop in GT335 signal (Fig. 4C). Inhibition of the elongating polyglutamylases TTLL1 or TTLL11, which resulted in a dramatic drop in the polyE signal without perturbation of the polyglutamate chain branching (GT335 signal), similarly caused a loss of septin in the MT fraction and along MTs (Fig. 4B and 4C), and a severe disorganization of septin filaments as well (Fig. 4C). Consistent with these findings, the overexpression of CCP1 (which is also overexpressed in Tr cells, see Fig. S2) or CCP5, both yielded a drop in septin recruitement to MTs (Fig. 4B and 4D) that was not related to any MT loss (Fig. 4D). Interestingly, both the impairments of polyglutamate side-chain formation or of chain elongation resulted in the restoration of Tr cell sensitivity to 10 nM Taxol^®^ (Fig. 4E left panel). Overexpression of the deglutamylation enzymes also shifted the viability curves but to a lesser extent (Fig. 4E right panel). Therefore, the level of MT polyglutamylation impacts the recruitment of septins to the MT lattice and both are important to confer taxane resistance.

### Septins enhance the recruitment to microtubules of enzymes that control the length of tubulin polyglutamylate chains

**Figure 5:**
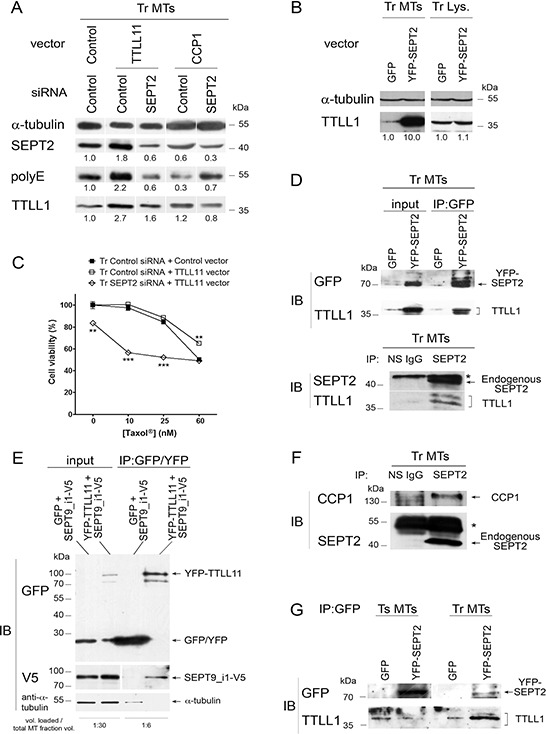
Microtubule-bound septins function as scaffolds to recruit tubulin polyglutamylation enzymes **A.** The overexpression of polyglutamylation (TTLL11) or deglutamylation (CCP1) enzyme combined or not with SEPT2 depletion modulates tubulin long chain polyglutamylation and alters the recruitment of the elongating enzyme TTLL1 to the MT fraction of Tr cells. **B.** The overexpression of SEPT2 boosts the recruitment of TTLL1 to MTs in Tr cells. Immunoblot analysis of MT fractions (Tr MTs) or whole cell lysates (Tr Lys.) was performed from cells subjected to the overexpression of YFP-SEPT2 or GFP alone as indicated. **C.** Preventing the recruitment of polyglutamylation enzymes to the MTs of Tr cells by SEPT2 RNAi restores chemosensitivity to Taxol^®^, even after TTLL11 overexpression. **D.** TTLL1 co-immunoprecipitates with SEPT2. Top panel: MT fractions from cells overexpressing YFP-SEPT2 or GFP alone were subjected to immunoprecipitation with anti-GFP/YFP antibody and analyzed for their contents in YFP-SEPT2 and TTLL1 as indicated. Bottom panel: the precipitation of endogenous SEPT2 from MT fractions allows the recovery of TTLL1. Non-specific antibody (NS IgG) was used as a control. The (*) indicates the antibody heavy chains used for precipitation. **E.** MT fractions from cells overexpressing either GFP or YFP-TTLL11 together with SEPT9_i1-V5 were subjected to immunoprecipitation with anti-GFP/YFP antibody and analyzed for their contents in GFP/YFP, YFP-TTLL11, SEPT9_i1 (as revealed by an antibody to the V5 tag) and α-tubulin as indicated. The volume loaded onto gels compared to the initial MT fraction volume is indicated at the bottom. **F.** CCP1 co-immunoprecipitates with SEPT2 in the MT environment of Tr cells. **G.** TTLL1 co-precipitation with YFP-SEPT2 from MT fractions only occurs in Tr cells.

Septin association with MTs has no impact on the presence of short polyglutamate chains, but is involved into chain elongation (see Fig. 3C) and hence into the chemoresistance process. As SEPT7 was evidenced to interact with the tubulin deacetylase HDAC6 in cell lysates [46], we thus wondered whether septin filaments could impact other tubulin-modifying enzymes by functioning as scaffolds to recruit elongating polyglutamylases and deglutamylases to MTs that already bear short glutamate side chains. First, we tested whether the recruitment and the function of elongating polyglutamylases and deglutamylases to MTs could be favored by the presence of septin filaments on MTs. As TTLL1 belongs to a complex and cannot be overexpressed alone, we chose to overexpress TTLL11 and to monitor the recruitment of endogenous TTLL1 to MTs. In Fig. 5A, we overexpressed YFP-tagged TTLL11 with or without septin filament dissociation by SEPT2 RNAi. While the stimulation of polyglutamate chain elongation (polyE signal) by TTLL11 overexpression enhanced both SEPT2 and TTLL1 recruitment to the MTs of Tr cells, polyglutamate chain elongation and TTLL1 recruitment were much less stimulated when cells were partially depleted of SEPT2 (Fig. 5A). Symmetrically, the overexpression of YFP-tagged SEPT2 highly increased TTLL1 recruitment to MTs (Fig. 5B). Also, in the absence of septin filaments, Taxol^®^ resistance of Tr cells was reversed, independently of TTLL11 expression level (Fig. 5C). Note that in Tr cells, even though tubulin long chain polyglutamylation level can be boosted by TTLL11 overexpression, resistance to Taxol^®^ cannot increase to a great extent (Fig. 5C). Together, these results indicate that septins function upstream of elongating TTLLs to enhance their recruitment to MTs. In a similar way, septins favor the binding to MTs of deglutamylases such as CCP1 to shorten polyglutamate chains. Indeed, CCP1 overexpression was much less effective to trim long glutamate chains when SEPT2 was depleted (Fig. 5A).

That septins could function as scaffolds for recruiting glutamate chain elongating and trimming enzymes was further evidenced by co-immunoprecipitation experiments performed on MT fractions of Tr cells. The immunoprecipitation of YFP-tagged SEPT2 (Fig. 5D, Top panel) or that of endogenous SEPT2 (Fig. 5D, Bottom panel) both allowed the co-precipitation of TTLL1. Symmetrically, V5-tagged SEPT9_i1 was co-precipitated with YFP-tagged TTLL11 that was captured on anti-GFP/YFP antibody-coated beads (Fig. 5E). By overexpressing SEPT9_i1 that has been shown to bind MTs *in vitro*, which is not the case for SEPT2, we tested whether co-precipitation could be mediated by tubulin. As shown in the bottom gel of Fig. 5E, when SEPT9_i1 was overexpressed together with TTLL1, tubulin recovery after precipitation was almost undetectable compared to the amount loaded (see the sample volume ratios indicated), indicating that co-precipitation did not result from the interaction of distant partners via tubulin. As the length of polyglutamate chains results from a balance between elongating TTLLs and trimming CCPs, we also checked that CCP1 co-precipitated with endogenous SEPT2 (Fig. 5F). Consistent with the above results, Fig. 5G further shows that the immunoprecipitation of YFP-tagged SEPT2 pulled down TTLL1, only when it was overexpressed in Tr but not in Ts cells (even though the level of YFP-SEPT2 expression was higher than in Tr), indicating that this scaffolding role of septins only exists when septin filaments are associated with MTs.

Altogether, these results show that MT-associated septin filaments function to enhance the recruitment of elongating polyglutamylases and glutamate chain trimming enzymes and thus participate in the control of polyglutamate chain elongation. In keeping with the scaffolding roles of septins on membrane compartments and actin stress fibers, we provide a new evidence of such a function on MTs.

### Tubulin retyrosination and/or the level of Tyr-tubulin control both septin recruitment to microtubules and the extent of microtubule polyglutamylation

**Figure 6:**
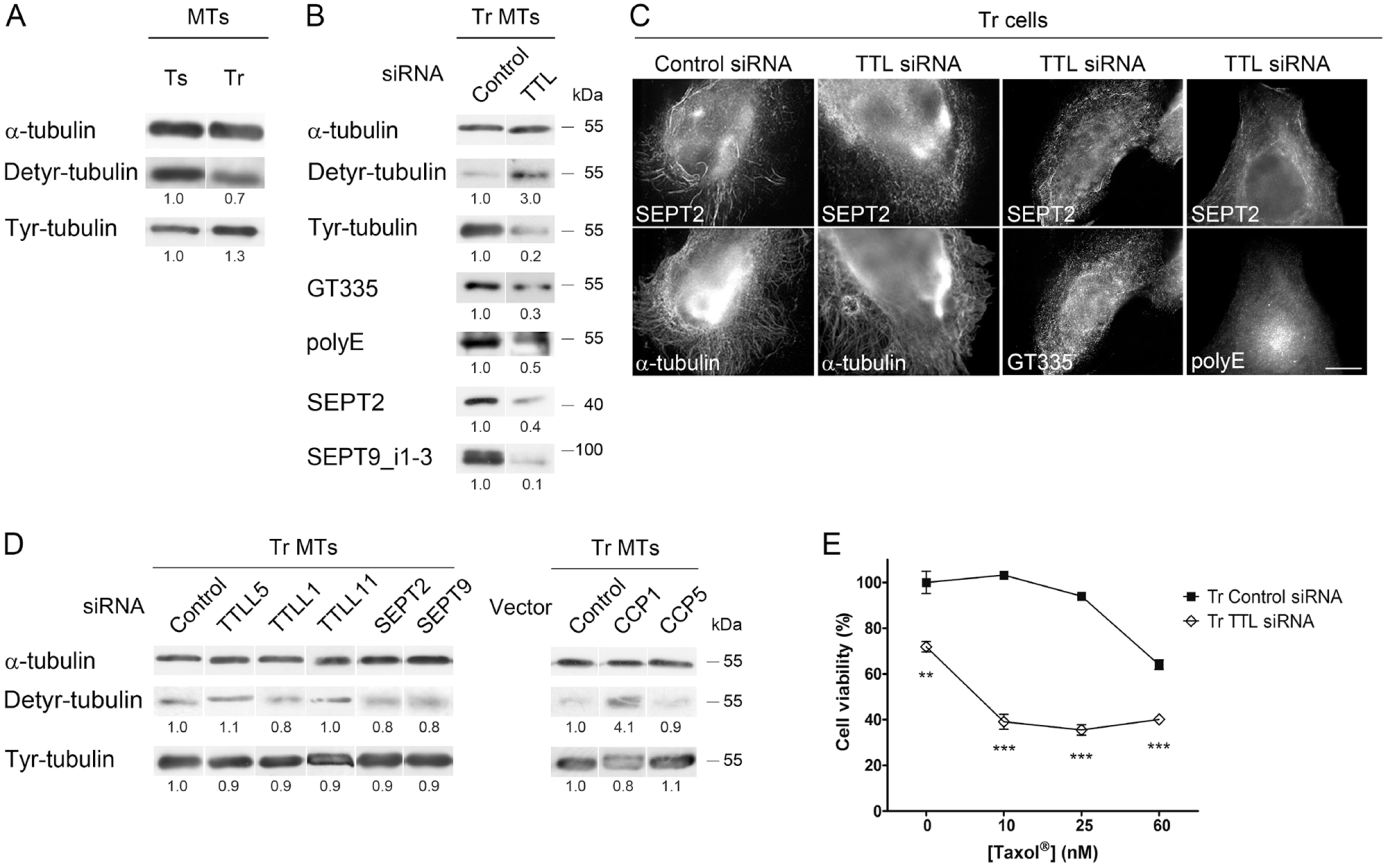
The level of Tyr-tubulin positively controls polyglutamylation and the recruitment of septins to microtubules, and therefore participates in Taxol^®^ resistance **A.** The MTs of Tr cells exhibit higher levels of Tyr-tubulin and lower levels of Detyr-tubulin than those of Ts cells. **B and C.** Inhibition of tubulin retyrosination by TTL RNAi alters MT polyglutamylation and septin recruitment to the MTs of Tr cells without affecting MT organization. Scale bar = 10 μm. **D.** Effect of the inhibition of tubulin polyglutamylation (by TTLL RNAi or CCP overexpression) or that of septin filament formation (by SEPT RNAi) on the Tyr/Detyr-tubulin balance in the MTs of Tr cells. **E.** TTL depletion by RNAi in Tr cells restores chemosensitivity.

From the above results, it appears that septin and polyglutamylation depend on each other. We thus asked if an upstream event could initiate this interplay at the MT level. Evaluation of the level of tubulin detyrosination/retyrosination in Ts and Tr cells revealed an imbalance between the Detyr- and Tyr-tubulin contents in the MT fractions of both cell lines (Fig. 6A). This imbalance is likely to be due to a higher expression of TTL in Tr *vs* Ts cells (Fig. S2). Surprisingly, knocking down TTL by RNAi in Tr cells (which led to an expected drop in Tyr- and an increase in Detyr-tubulin) strongly affected global tubulin polyglutamylation (as detected with both the GT335 and polyE antibodies) as well as the recruitment of septins (SEPT2 and SEPT9_i1–3) to MTs (Fig. 6B). Accordingly, double labeling immunofluorescence experiments revealed that septin organization into filaments was strongly affected by TTL knockdown and that MTs bearing long polyglutamate chains could no longer be detected in this condition (Fig 6C).

To confirm that the control of the Detyr-/Tyr-tubulin balance actually occurs upstream of the polyglutamylation/septin loop, we studied the effect of polyglutamylases, deglutamylases or septins on the tubulin detyrosination/retyrosination cycle in Tr cells. Neither knocking down TTLL5 (an initiating enzyme), TTLL1 or TTLL11 (both elongating enzymes), SEPT2 or SEPT9, nor overexpressing CCP5 impacted to a great extent the Detyr-/Tyr-tubulin balance (Fig. 6D). Unlike that of CCP5, the overexpression of the debranching enzyme CCP1 increased the Detyr-/Tyr- ratio in Tr cells (Fig. 6D), suggesting as already proposed in a previous study [47] that CCP1 could also function as a tubulin tyrosine carboxypeptidase in conditions of overexpression, although such an activity has not been detected *in vitro* [27].

Regarding chemoresistance, although TTL inhibition in Tr cells affected cell viability in the absence of Taxol^®^, it also very efficiently restored sensitivity to Taxol^®^ (Fig. 6E). From these results, we conclude that a high level of Tyr-tubulin is required to allow the initiation of tubulin polyglutamylation, septin recruitment to MTs and polyglutamate side-chain elongation.

### Tubulin modifications and septins are involved in CLIP-170 and MCAK recruitment to the microtubules of resistant cells

**Figure 7:**
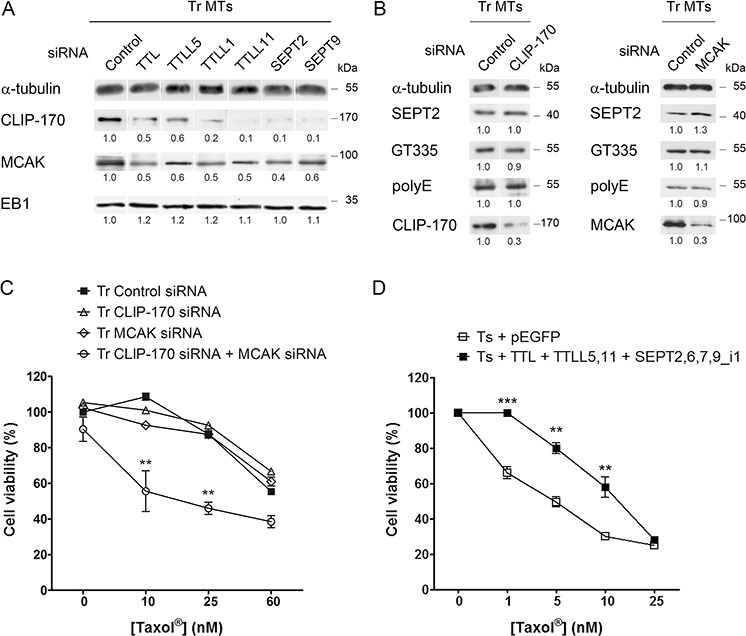
Tubulin modifications and septins control CLIP-170 and MCAK recruitment to the microtubules of Tr cells independently from EB1 **A.** Inhibition by RNAi of retyrosination or polyglutamylation enzymes or of septins alters CLIP-170 and MCAK recruitment to the MTs of Tr cells, but not that of EB1. **B.** Septin recruitment to the MTs of Tr cells and the level of polyglutamylated tubulin are not altered by the inhibition of CLIP-170 or MCAK. **C.** Contrasting with CLIP-170 or MCAK RNAi alone, the knockdown of both +TIPs restores chemosensitivity. **D.** Overexpression in Ts cells of TTL, TTLL5 (branching polyglutamylase), TTLL11 (elongating polyglutamylase) and of the components of Tr septin filaments makes cell resist ~10 nM Taxol^®^.

The rescue factor CLIP-170 and the depolymerase/catastrophe factor MCAK are recruited to the MT lattice via combined mechanisms that involve an interaction with EB1 [48] or with Tyr-tubulin [30, 31]. As the level of EB1 found on MTs does not markedly change between Ts and Tr cells (see Fig. 1E), we first tested whether the level of Tyr-tubulin, and/or the interplay between septins and MT polyglutamylation could explain the higher recruitment of CLIP-170 and MCAK to the MTs of Tr cells. As expected, the inhibition of tubulin retyrosination that resulted from TTL depletion caused an important loss of CLIP-170 and MCAK from the MT fraction of Tr cells while EB1 was only moderately affected (Fig. 7A). Similarly, TTLL5, TTLL1 and TTLL11 inhibition as well as SEPT2 or SEPT9 knockdown also resulted in CLIP-170 and MCAK loss while EB1 binding remained approximately constant. This result indicates that, in the context of cell adaptation to taxanes, the binding of CLIP-170 and MCAK to MTs would not only rely on the presence of high levels of Tyr tubulin, but also on the presence of septins on MTs and of long chain-polyglutamylated tubulin.

To finally ascertain that the modulation of CLIP-170 and MCAK binding to MTs occurred downstream of tubulin retyrosination and then of septin binding and tubulin polyglutamylation, we knocked down each +TIP by RNAi (Fig. 7B). Either CLIP-170 or MCAK depletion alone did not cause a decrease in tubulin polyglutamylation (either with short or long chains) or in septin recruitment to MTs. Furthermore, contrasting with the failure to recover Taxol^®^ sensitivity upon the separate inhibition of each +TIP, the dual knockdown of CLIP-170 and MCAK allowed Tr cells to recover sensitivity to Taxol^®^ (Fig. 7C). We may thus conclude that CLIP-170 and MCAK need to operate together, downstream of tubulin tyrosination, septin recruitment to MTs and tubulin long chain polyglutamylation to drive taxane resistance. By allowing more frequent catastrophes and rescues, they would contribute in sustaining high levels of MT dynamics to compensate for the MT-stabilizing effect of Taxol^®^. Indeed, the overexpression of all these partners in Ts sensitive cells, aiming at mimicking the different modifications exhibited by the resistant Tr cells, resulted in a significant increase in resistance to low concentrations of Taxol^®^ (Fig. 7D).

## DISCUSSION

**Figure 8:**
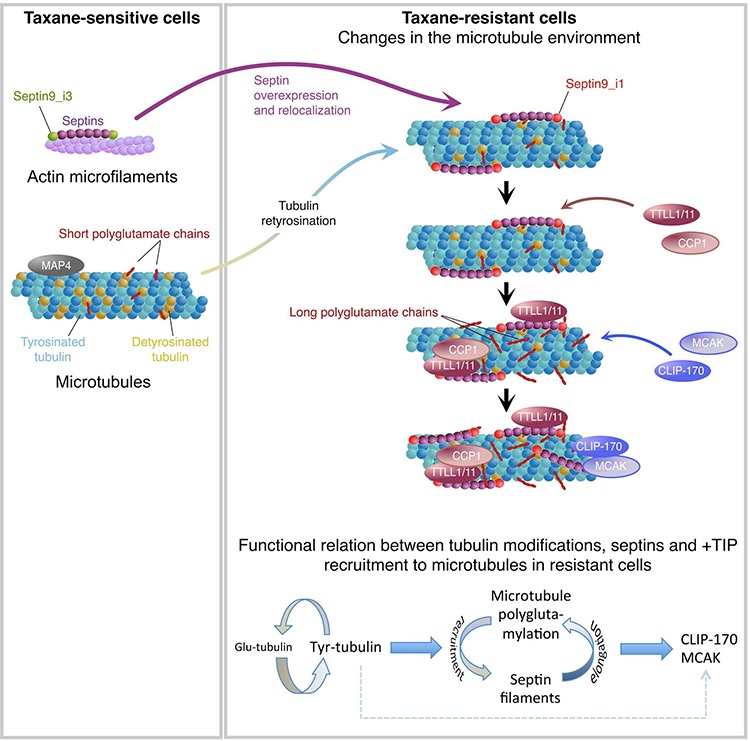
Integrative model of the molecular events that take place in the microtubule environment of taxane-adapted MDA-MB 231 cells

Taxanes are effective chemotherapeutic agents for treating patients with metastatic breast cancer or other advanced solid tumors [49], but their clinical use is limited by the emergence of drug resistance, which is a multifactorial process [2]. In a former study, we found that Taxol^®^-sensitive (Ts) or -resistant (Tr) MDA-MB 231 breast cancer cells display important differences in septin expression and recruitment to MTs as well as in the pattern of β-tubulin isotype expression [3]. Now, we demonstrate that septins participate in a mechanism of taxane resistance integrating both overexpression and increased recruitment to MTs of SEPT2, 7, 8, 9 and 11 and the MT enrichment in Tyr- and in long chain-polyglutamylated tubulin (Fig. 8). We evidenced that high levels of Tyr-tubulin facilitate the association of septin filaments containing the SEPT9_i1 isoform with MTs bearing short polyglutamate chains. MT-bound septins then enhance the binding of elongating polyglutamylases (TTLL1 or TTLL11) and trimming deglutamylase (CCP1) to the MT lattice to regulate polyglutamate chain elongation. MTs bearing septin filaments and long polyglutamate chains would then stimulate the binding of the rescue factor CLIP-170 and of the depolymerizing kinesin MCAK, which are critical to confer taxane resistance together. Among different MAPs, MCAK [50] and CLIP-170 [51, 52] have been proposed to modulate cancer cell sensitivity to Taxol^®^ [53] due to adaptive increase in MT dynamics [54]. Along with a reduced binding of the MT stabilizers MAP4 and survivin ([55], this study), higher CLIP-170 and MCAK levels would respectively promote rescues and catastrophes to stimulate MT dynamics, giving rise to the resistant phenotype observed in Tr cells.

### Septin isoform expression, microtubules and cancer

The ubiquitously expressed SEPT9 and its variant isoforms have been linked to breast, ovarian, prostate, colon, and head and neck cancers (for review, see Stanbery and Petty [56]). SEPT9 overexpression was first detected in ovarian tumors due to upregulation of *SEPT9_v1* and SEPT9_v4* transcripts [57]. In Tr cells, we actually detected more *SEPT9_v1* transcripts but SEPT9_v4* was not overexpressed (Figure S2). SEPT9_i1 overexpression is known to control the HIF-1α pathway [58, 59] and to stabilize the c-Jun N-terminal kinase [60], leading to enhanced proliferation and tumor growth. SEPT9_i1 expression has also been linked to cell resistance to paclitaxel [22] and it has been proposed to be associated with late cancer stages and poor outcomes [61, 62].

The high level of SEPT9_i1 could explain the striking change in septin filament location from actin (in Ts cells) to MTs (in Tr cells) upon adaptation to Taxol^®^. Septin filaments actually colocalize with actin in almost all non-dividing cells, and with MTs only in a few cases (for review see Spiliotis [63]). By directly interacting *in vitro* with MTs through its GTP-binding domain [5], SEPT9_i1 would promote the association of septin filaments with MTs in interphase cells [64] and during cytokinesis in mammalian cells [64, 65]. Septins were proposed to associate with bundled rather than individual MTs [66, 67]. In this respect, Taxol^®^-induced bundling of MTs would increase septin affinity [68] but at 25 nM, we could hardly detect such an effect on the MTs of Ts cells (Fig. S1). Repeated motifs in the N-terminal domain of the long SEPT9 isoforms (SEPT9_i1 and SEPT9_i3) would in fact bind to and bundle MTs by interacting with the acidic C-terminal tail of β-tubulin, as shown in MDCK cells [23]. Nevertheless, the difference of septin filament localization between our Ts and Tr cells cannot originate from the presence/absence of these motifs as both cell lines express long SEPT9 isoforms (SEPT9_i3 in Ts and _i1 in Tr).

### Tubulin tyrosine ligase expression and function

SEPT2 binding to MTs was proposed earlier to influence their level of polyglutamylation [14]. We now show that a high level of Tyr-tubulin and/or of TTL, the expression of which was actually higher in Tr *vs* Ts cells (Figure S2), functions in close relationship with septin recruitment to the MT lattice and MT polyglutamylation level to confer Taxol^®^ resistance. Indeed, preventing tubulin retyrosination by knocking down TTL severely impaired the binding of septin filaments to MTs and the parallel recruitment of the molecular machinery that controls tubulin polyglutamate chain length.

Detyrosination of α-tubulin negatively regulates the binding of CLIP-170 and MCAK to the MT plus ends [30, 31], thereby suggesting a mechanism to explain the shorter lifetime and the higher levels of dynamic instability of the MTs of Tr cells compared to those of Ts exposed to short-term Taxol^®^ treatment (Fig. 1D). However, CLIP-170 and MCAK binding to the MTs of our taxane-adapted cells also requires septins and/or long chain polyglutamylation, indicating that a high level of Tyr-tubulin is necessary but not sufficient to stimulate MT dynamics in our model. Tyr-tubulin incorporation into growing MTs has never been shown to stimulate alone polyglutamylation and septin recruitment, suggesting that TTL would play a role in these regulations beside of its enzyme activity. Also, contrasting with the acceleration of MT polymerization we observed in Tr cells, overexpressed TTL (see Fig. S2) would be expected to slow MT growth [69]. As this slowdown occurs via the sequestration of free tubulin, we could speculate that MT-bound septins could facilitate the release of TTL from the Tyr-tubulin/TTL complex.

Another intriguing question raised by our data comes from the contrast between decreased TTL expression (and thus high levels of Detyr-tubulin) observed in aggressive mammary and prostate tumors [28, 70] and the high Tyr-tubulin levels found in paclitaxel-resistant MCF-7 breast cancer cells [29] and in our taxane-adapted cells (this study). Although it is clear that this high level of Tyr-tubulin is essential to confer taxane resistance (Fig. 6), whether it results only from high retyrosination (increased TTL) or also involves decreased detyrosination will deserve further investigation.

### Control of the extent of tubulin polyglutamylation

The length of the glutamate chain fine tunes the binding of MAPs to MTs. Indeed, tau, MAP2 and kinesin prefer tubulin with ~3 glutamate units while MAP1A or the severing enzyme spastin prefer 6-unit long chains [24, 25, 71, 72]. Here, by modulating glutamate chain length (via the control of TTLL or CCP expression), we show that long chain tubulin polyglutamylation is essential for the acquisition of Taxol^®^ resistance. Increased TTLL12 (an elongating polyglutamylase) has already been associated with metastatic progression of prostate cancer [73]. Furthermore, as it has already been observed in a prostate cancer cell line resistant to estramustine [74], polyglutamylation might be a more general feature in acquired resistance to anti-tubulin agents.

Our data point out the key role of septins in the control of glutamate chain length as they would favor the binding of elongating TTLLs and trimming CCPs to increase and maintain long polyglutamate side chains (Fig. 8). A scaffolding function of septins was formerly described in yeast during cytokinesis completion but it also applies to other cell compartments during the interphase (see review of Beise and Trimble [75]). For example, septin-mediated scaffolding of myosin II and of its kinases occurs on actin stress fibers for full myosin activation [76]. Septins also maintain the cytoplasmic localization of SOCS7 and NCK to control actin organization at steady state; the loss of this compartmentalization and the nuclear translocation of these factors being required for DNA damage response [77]. We now show that septins also function on MTs as dynamic regulators of tubulin polyglutamylation via TTLL and CCP recruitment. As proposed for their scaffolding function in budding yeast during cytokinesis [78], septin high order of organization around MTs might enhance polyglutamylation enzyme recruitment on MTs even without a direct interaction. This notion is in keeping with the finding that TTLL6 targeting to cilia also needs CEP41 as a scaffolding protein [79]. Along with high levels of Tyr-tubulin, a control of the length of polyglutamate chains on tubulin by septins would in turn drive the recruitment to the MT lattice of regulators of MT dynamics.

Regarding Taxol^®^ resistance, we are now more precisely investigating whether TTL, TTLL5 or 11 and septins, among which SEPT9_i1 or_i3, alone or in combination, can induce Taxol^®^ resistance and whether these changes of expression allow the shift of septin filaments from actin to MTs in MDA-MB 231 cells but also in other cancerous cell types.

In conclusion, understanding cancer cell resistance to taxane chemotherapy is still a heavy burden to improve the effectiveness of anticancer treatments. The relevant localization of septin hetero-oligomers on MTs of taxane-resistant cells supports the significance of septins, in particular of SEPT9_i1, as well as that of tubulin tyrosination and polyglutamylation (and of their respective enzymatic machinery) as effectors of breast cancer pathogenesis and hallmarks of tumor cell adaptation to chemotherapy. This also opens the exciting possibilities that they could be used as new tools for diagnosis, prognosis and even for suggesting new therapeutic strategies in the management of breast cancer.

## MATERIALs AND METHODS

### Cell culture and viability assays

The Taxol^®^-resistant (Tr) and -sensitive (Ts) sublines of the human breast carcinoma cell line MDA-MB 231 were established by progressively increasing Taxol^®^ (Sigma-Aldrich) concentrations and continuously cultured without (Ts) or with 25 nM Taxol^®^ (Tr) as previously described [3]. Sensitivity to Taxol^®^, Taxotere^®^, Epothilone B, Colchicine, Vinblastine or Cisplatin (Sigma-Aldrich) was measured using 3-(4,5-Dimethylthiazol-2-yl)-2, 5-diphenyltetrazolium bromide (MTT) cytotoxicity assay after 48 h exposition. The IC_50_ was then determined with Prism4 software (GraphPad software). Each value represents the mean ± s.e.m. of at least 3 independent experiments.

### Flow cytometry cell cycle analysis

Cell cycle analysis was performed as previously described [80] on 3 independent Ts and Tr cell samples with or without 25 nM Taxol^®^ for 24 h. The DNA content was analyzed by flow cytometry using FACS Fortessa controlled by FACSDiva software (BD Biosciences), and cell cycle analysis was performed using FlowJo software (Tree Star, Inc.).

### Plasmid and siRNA transfections

Plasmid transfections were performed using Lipofectamine™ LTX (Invitrogen) according to the manufacturer's instructions with an expression vector concentration of 1 μg/mL, 48 h prior to analysis. pEGFP (Addgene) was used as a negative control. CFP-CCP1, CFP-CCP5, CFP-TTLL5 and YFP-TTLL11 expression vectors were kind gifts of Dr. C. Janke (Institut Curie, Orsay, France). YFP-SEPT2 and SEPT9_i1-V5 were kindly provided by Dr E.T. Spiliotis (Drexel University, Philadelphia, PA, USA) and Dr. A. Gassama (CHB Paul Brousse, Villejuif, France), respectively.

Short interfering RNA (siRNA) transfections were made using the calcium phosphate method 72 h prior to analysis. For SEPT2, SEPT9, TTL and TTLL1, two different siRNAs were tested for the silencing of target genes. The sequences of all used siRNAs (Sigma-Aldrich or Santa-Cruz Biotechnology) are listed in Table S1. A nonsilencer siRNA (D-001810–10-50, Dharmacon) was used as a negative control. When necessary, cells transfected with siRNA (control or SEPT2) were grown 24 h before plasmids encoding CFP-CCP1 or YFP-TTLL11 were transfected.

### Immunoblotting

Total cells lysates and MT fractions were prepared as previously described [3]. Constant protein amounts (10 μg) were loaded onto gels for total cell lysate analysis, while constant α-tubulin amounts were used for MT fraction analysis. After 9% SDS-PAGE separation, transfer onto PVDF membranes and saturation with 10% non-fat dry milk, blots were probed with selected primary antibodies against: α-tubulin (clone DM1-A) and acetylated-α-tubulin (Sigma-Aldrich), Detyr-tubulin (AbCys), SEPT2 and 11 (Atlas), SEPT7 (H120), SEPT8 (N15), EB1 (H70), CLIP170 (H300) and Survivin (Santa-Cruz Biotechnology), MCAK (Abnova), MAP4 (BD Biosciences), TTLL1 (AbCam), GFP (Cell Signaling), V5 (Invitrogen). Anti-Tyr-tubulin (clone YL1/2), anti-polyglutamylated-tubulin (clone GT335) and anti-SEPT9_i1, 3 and 4 antibodies were kindly provided by Dr L. Lafanechère (Institut Bonniot, Grenoble, France), Dr P. Denoulet (UPMC, Paris, France) and Pr P. Cossart (Institut Pasteur, Paris, France), respectively. The anti-long chain-polyglutamylated-tubulin (polyE) antibody was prepared and characterized by Drs C. Janke (Institut Curie, Orsay, France) and T. Surrey (London Research Institute, London, England). Protein bands were visualized with respective HRP-conjugated secondary antibodies and the ECL detection kit (Pierce). Western-blot quantification was performed after film digitization using the ImageJ software (http://imagej.nih.gov/ij/). All the quantitative data are the ratio of the intensity of the protein of interest to that of α-tubulin for the blot shown, which is representative of the blots obtained from at least 3 independent experiments.

### Immunoprecipitation

Immunoprecipitation experiments were performed on MT fractions that were obtained as described in Froidevaux-Klipfel et al. [3]. It is to note that after plasma membrane solubilisation and cytosol removal in a MT-stabilizing buffer, MTs were extensively depolymerized by calcium upon extraction. Thus, the MT fraction contains the MT-associated proteins and the formerly polymerized tubulin subunits. Immediately after fractionation or two days after appropriate cell transfections, MT fractions were incubated with rabbit anti-SEPT2 antibody or rabbit non-specific serum-coated protein G sepharose beads (GE Healthcare), or with anti-GFP/YFP-coated beads (GFP Trap^®^_M kit, Chromotek) in a buffer containing 10 mM Tris pH 7.5, 150 mM NaCl, 5 mM EDTA, 1% CHAPS and a mixture of protease and phosphatase inhibitors. After constant rotation at 4°C for 16 h, the beads were washed four times in ice-cold wash buffer (10 mM Tris pH 7.5, 150 mM NaCl, 5 mM EDTA) with the salt concentration increased to 300 mM in the last two washing steps. Protein samples were then boiled in Laemmli buffer before immunoblot analysis.

### Immunofluorescence and microscopy

Cells grown on glass coverslips were immunostained with primary antibodies as indicated or TRITC-conjugated phalloidin (Sigma-Aldrich) to probe actin, after fixation with 3.75% paraformaldehyde/PBS, permeabilization with 0.05% Saponin/PBS and blocking with 0.5% BSA/PBS. Secondary antibodies were Alexa Fluor 488- or 555-conjugated antibodies against rabbit or mouse IgGs (Molecular Probes). For live MT tracking and dynamic instability measurements, cells grown on glass coverslips were transfected with GFP-DDA3 (kindly provided by Dr G. Fang, Stanford University, CA, USA). Coverslips were mounted in a microchamber and maintained at 37°C using a PeCon on-stage thermal controller.

Images or time-lapse sequences were acquired using a Scion CFW1312M CCD camera on a Leica DMLB microscope (100x 1.3 NA objective), driven from an Apple iMac computer and homemade software.

### Quantification and analysis of microtubule dynamics

The quantification and analysis of MT dynamics was performed as previously described [81]. The duration, extent and rate for a selected phase were determined by a linear regression of life history plots in Microsoft Excel. The mean duration, extent, growth and shrinking rates and the average duration of pause and lifespan resulted from the tracking of a minimum of 8 MTs from at least 3 cells in each condition. Catastrophe and rescue frequencies were calculated as described in [82].

### Statistical analysis

Quantitative data are the means ± s.e.m. of at least three independent experiments. Taxol^®^ cytotoxicity measurements and MT dynamic instability parameters were compared using Student's *t* test. The following symbols were used: * means *p* < 0.05, ** means *p* < 0.01, and *** means *p* < 0.001.

## SUPPLEMENTARY MATERIALS AND METHODS FIGURES AND TABLE


