# Supplementary material for: Septin cooperation with tubulin polyglutamylation contributes to cancer cell adaptation to taxanes
Source: Oncotarget. 2015 Oct 7;6(34):36063–80. doi: 10.18632/oncotarget.5373 (PMC4742162; doi:10.18632/oncotarget.5373)
Supplement: Supplementary file 1 [file oncotarget-06-36063-s001.pdf]

SUPPLEMENTARY MATERIALS AND METHODS

2D-WESTERN BLOT ANALYSIS

For 2D-Western blotting, 100 µg of proteins from MT fractions were subjected to isoelectrofocalisation using linear pH4–7 strips (GE-Healthcare) prior to second dimension electrophoresis, as described previously [3]. SDS-PAGE, transfer and immunodetection were performed as described in the experimental procedure section.

RT-PCR ANALYSIS

Total RNA was isolated using Extract-ALL phenol-guanidine reagent (Eurobio) and chloroform, precipitated with isopropanol, and was reverse transcribed with SuperScriptIII (Life Technologies) according to the manufacturer's instructions. Real-time PCR was performed with SYBR Green (Applied Biosystems) detection using primers specific for *SEPT9_v1* (forward CGGAGGCACCATGAAGAAGT, reverse GGGCCACTGGAGTCACCAA), *SEPT9_v4** (forward GGCAGGAGTGTCATCTTTTC, reverse GCGTGCCTCCTGGACA), *TTLL1* (forward GGTGCTCTGTCAGTGCCTATG, reverse TGCACACTCATCCAGTAAAAATTC), *TTL* (forward GAGGCTTCAGAGCTTCTCGA, reverse CCAGCTTCGGATGTCAAACT) and *CCP1* (forward GCTGCCCCTTGGTGACTATA, reverse TTCTCCAGGATGTACCCGAG). Relative gene expression is the ratio of the mRNA content of target genes to those of human *GAPDH* (forward CAGCCTCAAGATCATCAGCA, reverse TGTGGTCATGAGTCCTTCCA), 18S (forward TGAGAAACGGCTACCACATC, reverse TTACAGGGCCTCGAAAGAGT) and TBP-H2 (forward GAGAGCCACGAACCACGG, reverse ACATCACAGCTCCCCACCAT) house-keeping genes, according to the ΔΔCt method [84]. Reverse and forward oligonucleotide primers, specific to the chosen candidate and housekeeping genes, were designed using Primer3 software (http://frodo.wi.mit.edu/cgi-bin/primer3/primer3_www.cgi).

REFERENCES

84. Livak KJ. Analysis of relative gene expression data using real-time quantitative PCR and the 2) Method. Methods. 2001; 25:402–408.

85. Spiliotis ET, Kinoshita M, Nelson WJ. A mitotic septin scaffold required for Mammalian chromosome congression and segregation. Science. 2005; 307:1781–1785.

86. Montagna C, Lyu MS, Hunter K, Lukes L, Lowther W, Reppert T, Hissong B, Weaver Z, Ried T. The Septin 9 (MSF) gene is amplified and overexpressed in mouse mammary gland adenocarcinomas and human breast cancer cell lines. Cancer Res. 2003; 63:2179–2187.

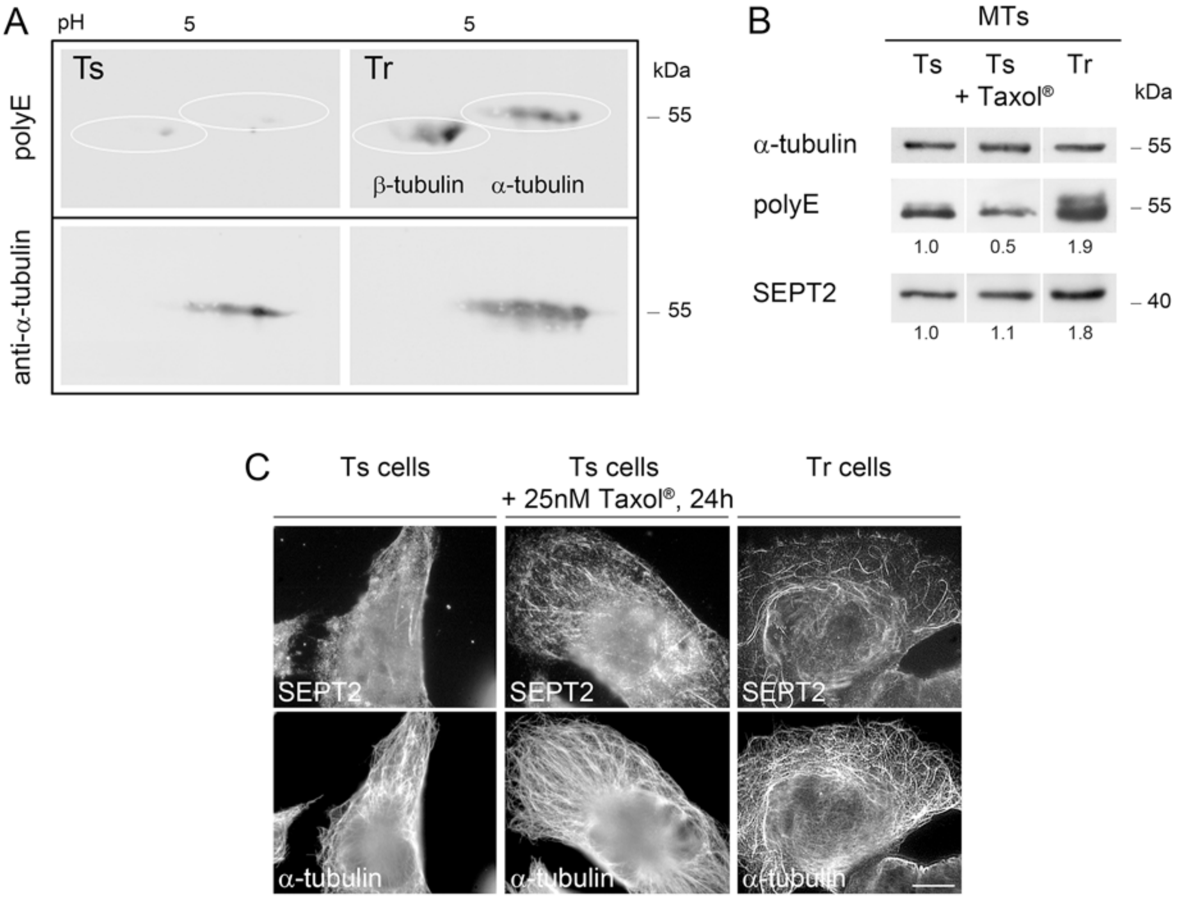

Supplementary Figure S1: A. Both α- and β-tubulin display long polyglutamylated chains in Tr cells. Levels of polyglutamylation (polyE signal) of α- and β-tubulin were analyzed by 2D-Western blotting on MT fractions of Ts and Tr cells. **B.** MTs of Ts cells treated with 25 nM Taxol® for 24 h do not exhibit a higher level of long chain polyglutamylated tubulin (polyE signal) and septin recruitment than Ts cells. **C.** Acute Taxol® treatment of Ts cells (25 nM Taxol®, 24 h) does not relocalize septins to MTs. Scale bar = 10 µm.

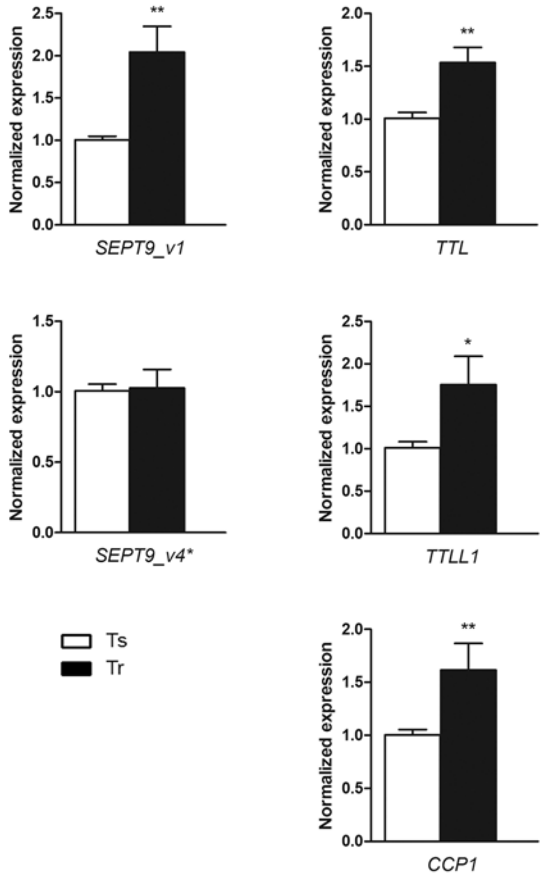

Supplementary Figure S2: Transcript expression of septins and polyglutamylation enzymes were evaluated by qPCR analysis in Ts and Tr cells. Histograms represent normalized transcripts expression.

Supplementary Table S1: siRNA used to deplete cells of septins, tubulin modification enzymes and +TIPs

siRNAs	*Suppliers*	*Sequences*
SEPT2 **1**	Sigma *CUSTOM oligos* [85]	*sense* 5′-AAGGUGAAUAUUGUGCCUGUC [dT][dT]-3′<br>*antisense* 5′-GACAGGCACAAUAUUCACCUU [dT][dT]-3′
SEPT2 **2**	Sigma *MISSION SASI_ Hs02_00305472*	*sense* 5′-GAAUGAGGACAUGAAUAAA [dT][dT]-3′<br>*antisense* 5′-UUUAUUCAUGUCCUCAUUC [dT][dT]-3′
SEPT9 **1**	Sigma *CUSTOM oligos* [86]	*sense* 5′-GUCCACUUUAAUCAAUACC [dT][dT]-3′<br>*antisense* 5′-GGUAUUGAUUAAAGUGGAC [dT][dT]-3′
SEPT9 **2**	Sigma *MISSION SASI_ Hs02_00325891*	*sense* 5′-GUGUGCAGAUCAUCCGUCU [dT][dT]-3′<br>*antisense* 5′-AGACGGAUGAUCUGCACAC [dT][dT]-3′
SEPT11	Sigma *CUSTOM oligos* [65]	*sense* 5′-CAAGAGGAAUUGAAGAUUAAA [dT][dT]-3′<br>*antisense* 5′-UUUAAUCUUCAAUUCCUCUUG [dT][dT]-3′
TTL **1**	Sigma *MISSION SASI_ Rn02_00198137*	*sense* 5′-CCAACUAAGAGGUCAUUAA [dT][dT]-3′<br>*antisense* 5′-UUAAUGACCUCUUAGUUGG [dT][dT]-3′
TTL **2**	Sigma *MISSION SASI_ Hs01_00198137*	*sense* 5′-GAGUUCAAUCAGUACCUAA [dT][dT]-3′<br>*antisense* 5′-UUAGGUACUGAUUGAACUC-3′
TTL **3**	Santa Cruz *(sc-94871)*	
TTLL1 **1**	Sigma MISSION SASI_ Hs01_00245872	*sense* 5′-GACAUCAUCAUCGACGACA [dT][dT]-3′<br>*antisense* 5′-UGUCGUCGAUGAUGAUGUC-3′
TTLL1 **2**	Santa Cruz *(sc76772)*	
TTLL5	Sigma *MISSION SASI_ Hs01_00168438*	*sense* 5′-GAAACUGUCUCGUCCCUCU [dT][dT]-3′<br>*antisense* 5′-UGUCGUCGAUGAUGAUGUC-3′
TTLL11	Sigma *MISSION SASI_ Hs01_00141865*	*sense* 5′-CAUCUACUGGCAUGGAGUU [dT][dT]-3′<br>*antisense* 5′-AACUCCAUGCCAGUAGAU-3′
CLIP-170	Sigma *CUSTOM oligos*	*sense 5′-GCACAGCUCUGAAGACACC [dT][dT]-3′*<br>antisense 5′-GGUGUCUUCAGAGCUGUGC [dT][dT]-3′
MCAK	Santa Cruz *(sc-105596)*	
